# Lycopodium Attenuates Loss of Dopaminergic Neurons by Suppressing Oxidative Stress and Neuroinflammation in a Rat Model of Parkinson’s Disease

**DOI:** 10.3390/molecules24112182

**Published:** 2019-06-10

**Authors:** Richard L. Jayaraj, Rami Beiram, Sheikh Azimullah, Mohamed Fizur Nagoor Meeran, Shreesh K. Ojha, Abdu Adem, Fakhreya Yousuf Jalal

**Affiliations:** Department of Pharmacology and Therapeutics, College of Medicine and Health Sciences, United Arab Emirates University, Al Ain, United Arab Emirates; richardlj@uaeu.ac.ae (R.L.J.); azim.sheikh@uaeu.ac.ae (S.A.); nagoormeeran1985@uaeu.ac.ae (M.F.N.M.); shreeshojha@uaeu.ac.ae (S.K.O.); fakhreya@uaeu.ac.ae (F.Y.J.)

**Keywords:** lycopodium, matrix metalloproteinase, neuroinflammation, neurodegeneration, oxidative stress, Parkinson’s disease

## Abstract

Parkinson’s disease, a chronic, age related neurodegenerative disorder, is characterized by a progressive loss of nigrostriatal dopaminergic neurons. Several studies have proven that the activation of glial cells, presence of alpha-synuclein aggregates, and oxidative stress, fuels neurodegeneration, and currently there is no definitive treatment for PD. In this study, a rotenone-induced rat model of PD was used to understand the neuroprotective potential of Lycopodium (Lyc), a commonly-used potent herbal medicine. Immunohistochemcial data showed that rotenone injections significantly increased the loss of dopaminergic neurons in the substantia nigra, and decreased the striatal expression of tyrosine hydroxylase. Further, rotenone administration activated microglia and astroglia, which in turn upregulated the expression of α-synuclein, pro-inflammatory, and oxidative stress factors, resulting in PD pathology. However, rotenone-injected rats that were orally treated with lycopodium (50 mg/kg) were protected against dopaminergic neuronal loss by diminishing the expression of matrix metalloproteinase-3 (MMP-3) and MMP-9, as well as reduced activation of microglia and astrocytes. This neuroprotective mechanism not only involves reduction in pro-inflammatory response and α-synuclein expression, but also synergistically enhanced antioxidant defense system by virtue of the drug’s multimodal action. These findings suggest that Lyc has the potential to be further developed as a therapeutic candidate for PD.

## 1. Introduction

Parkinson’s disease (PD), the second most common neurodegenerative disease following Alzheimer’s, affects 1–3% of population older than 50 [1,2]. This progressive neurodegenerative disease is characterized by selective loss of dopaminergic neurons in the Substantia nigra pars compacta and a subsequent decrease in dopamine levels in the striatum (caudate and putamen) [3,4].

Though the etiology of PD remains obscure, compelling experimental evidence suggests that oxidative damage, inflammation, mitochondrial dysfunction, endoplasmic reticulum stress and disturbed proteostasis to be the cardinal factors contributing to PD pathogenesis [5,6]. Histopathological examinations and post-mortem studies reveal the presence of intracytoplasmic eosinophilic proteinaceous inclusions, termed Lewy bodies, which are made up of aggregated alpha-synuclein, an active contributor to PD pathology [7,8]. Despite various treatment strategies, such as dopamine replacement therapy, monoamine oxidase inhibitors or dopamine receptor agonists, there is limited efficiency over the course of the disease, and these prolonged treatments often result in adverse effects whether motor (dyskinesia, motor fluctuation) and non-motor (sleep disorder, impulse control disorder, PD dementia) [9]. The etiopathogenesis of PD involves alteration in antioxidant defense and oxidative stress, following subsequent inflammation-mediated dopa-minergic neurodegeneration initiated by activation of microglia and astrocytes. Recent studies have identified potential neuroinflammatory factors, such as Matrix metalloproteinase (MMP) and α-synuclein as important modulators of microglia [10,11,12]. Hence, to counteract these, natural drugs with robust anti-oxidant and anti-inflammatory properties would help to guard dopaminergic neurons, and alleviate disease progression.

Considering the role of oxidative stress and neuroinflammation in inducing dopaminergic neuronal loss, the focus is on investigating the agents of natural origin which are gaining importance due to their health benefits, therapeutic potential and pharmacological properties. Many of the natural agents targeting oxidative stress and neuroinflammation have been shown beneficial in neurodegenerative diseases, including PD [13,14,15]. Hence, to counter oxidative stress and inflammation-mediated dopaminergic neurodegeneration, natural drugs with robust anti-oxidant and anti-inflammatory properties would help to protect dopaminergic neurons and alleviate disease progression.

In the present study, we evaluated the neuroprotective potential of Lycopodium using a rotenone-induced PD rat model. Although various agents such as reserpine, haloperidol, MPTP, paraquat, maneb and rotenone are used to induce pathological features of PD, rotenone, being an environmental toxicant, is one of the best models used to mimic the neuropathological and behavioral features of this disease. Rotenone has the ability to cross the Blood-brain barrier due to its lipophilic property, and that it selectively inhibits Complex I of the mitochondrial electron transport chain, resulting in the production of ROS, glutathione depletion and subsequent dopaminergic neurodegeneration. Moreover, rotenone administration results in induction of oxidative stress and neuroinflammation subsequent to activation of microglia, astrocytes and the inhibition of proteosomal activity, along with the accumulation of alpha-synuclein positive nigral inclusions, a hallmark of PD [16].

Lycopodium (syn. Huperzia) belongs to the family Lycopodiaceae, and it is also known as ground pine, club moss or devil’s claw (in English). It is found more in tropical and sub-tropical forests [17]. It has been a popular remedy in traditional Chinese and homeopathic medicines for centuries [18,19,20]. It has also been reported to possess anti-cancer [21,22], analgesic [23] and anti-inflammatory [24,25,26] properties. Lycopodium alkaloids, such as quinolizine or pyridine and α-pyridone-types, have been reported to possess acetylcholinesterase inhibition activity. Chemical, pharmacological and clinical research on Lycopodium alkaloids have been reviewed extensively elsewhere [27]. The whole plant extract has been reported to act as a CNS stimulant for motor anomalies in children [28]. However, there is no report available on its possible role in neuroprotection against neurodegeneration. Therefore, the present study was performed to evaluate the neuroprotective efficacy of Lyc against rotenone induced-neurodegeneration, a rat model of PD.

## 2. Results

### 2.1. Administration of Lyc Diminished Lipid Peroxidation and Enhanced Glutathione Levels in Rotenone Treated Rats

**Figure 1:**
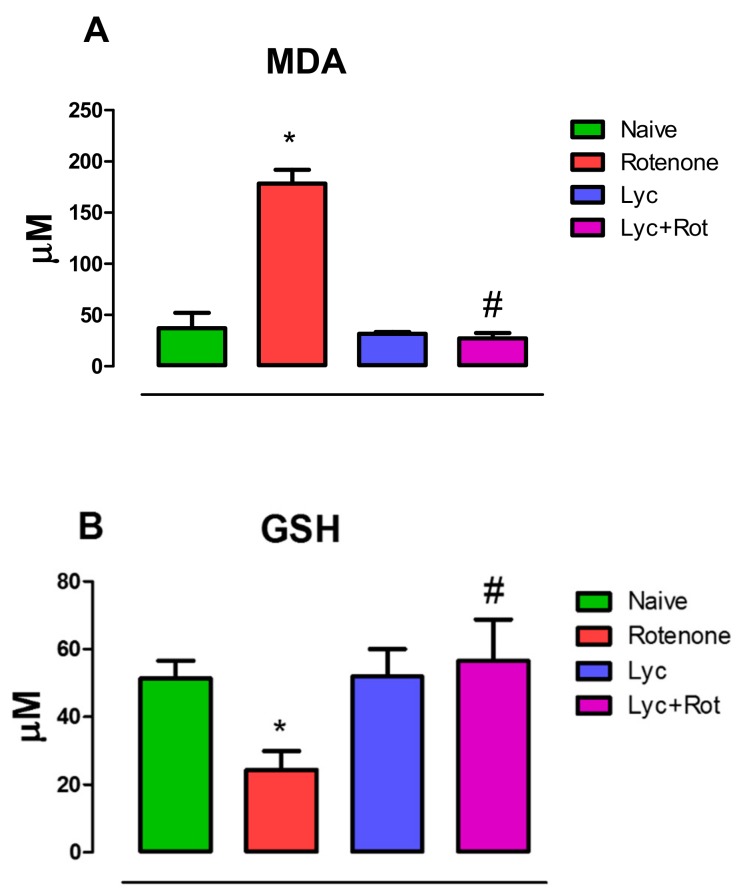
Effect of Lyc on Malonaldehyde and GSH content in the midbrain of rotenone-treated rats. Rotenone administration caused a significant increase (*p* < 0.05) in lipid peroxidation, which is represented by increase in malonaldehyde levels (**A**) with a marked decrease in GSH (**B**). However, treatment with Lyc prevented lipid peroxidation and enhanced glutathione levels. Values are expressed as mean ± SEM. * *p* < 0.05 compared to control, ^#^ *p* < 0.05 compared to rotenone treated group.

Lipid peroxidation assessment by measuring malondialdehyde levels (MDA) is one of the important biochemical markers for depicting the pathogenic event in causing oxidative stress-induced cellular impairment. Rotenone administration caused a significant (*p* < 0.05) increase in MDA levels in rotenone-injected rats, compared to the normal control (Figure 1A). Simultaneously, rotenone administration also caused significant (*p* < 0.05) reduction in GSH levels (Figure 1B), an antioxidant substrate in the glutathione redox cycle. Whereas, treatment with Lyc significantly (*p* < 0.05) attenuated lipid peroxidation and increased GSH levels compared to rotenone treated animals.

### 2.2. Lycopodium Reduced Rotenone-Induced Antioxidant Loss and Nitrite Content

**Figure 2:**
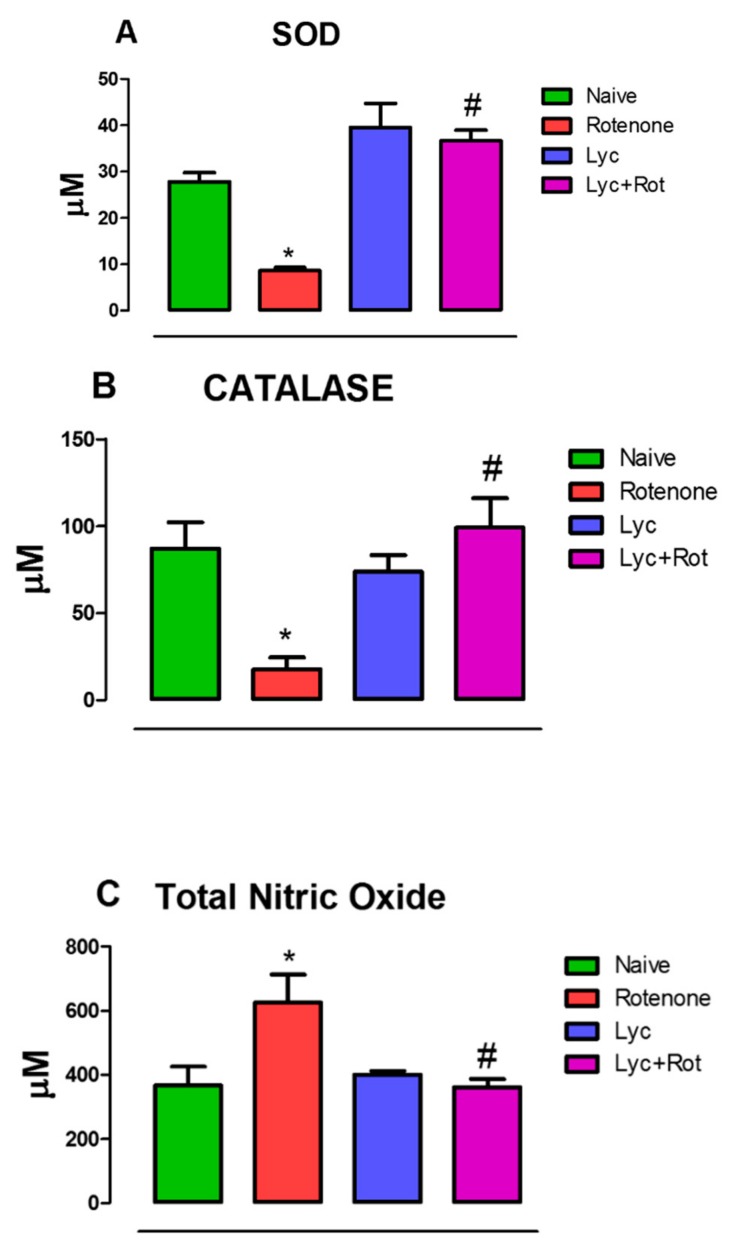
Evaluation of SOD, CAT and nitric oxide in experimental animals. Administration of rotenone-ameliorated brains vital antioxidant, SOD (**A**), CAT (**B**) with concomitant increase in total nitric oxide levels (**C**). Alternatively, Lyc treatment prevented nitric oxide production and reversed antioxidant loss. Values are expressed as mean ± SEM. * *p* < 0.05 compared to control, ^#^ *p* < 0.05 compared to rotenone treated group.

To determine whether lycopodium possess any antioxidant property, we assessed Superoxide dismutase (SOD) and Catalase (CAT) activities, which are key players in antioxidant defense system. Results showed that rotenone administration significantly (*p* < 0.05) enhanced oxidative stress, which is reflected by a significant decrease in SOD and CAT levels with marked increase (*p* < 0.05) in nitrite levels compared to control. In contrast, Lyc treatment substantially (*p* < 0.05) increased SOD (Figure 2A), CAT (Figure 2B) activities and decreased nitrite levels (Figure 2C) compared to rotenone-injected animals.

### 2.3. Lyc Mitigates Pro-Inflammatory Cytokines Expression

**Figure 3:**
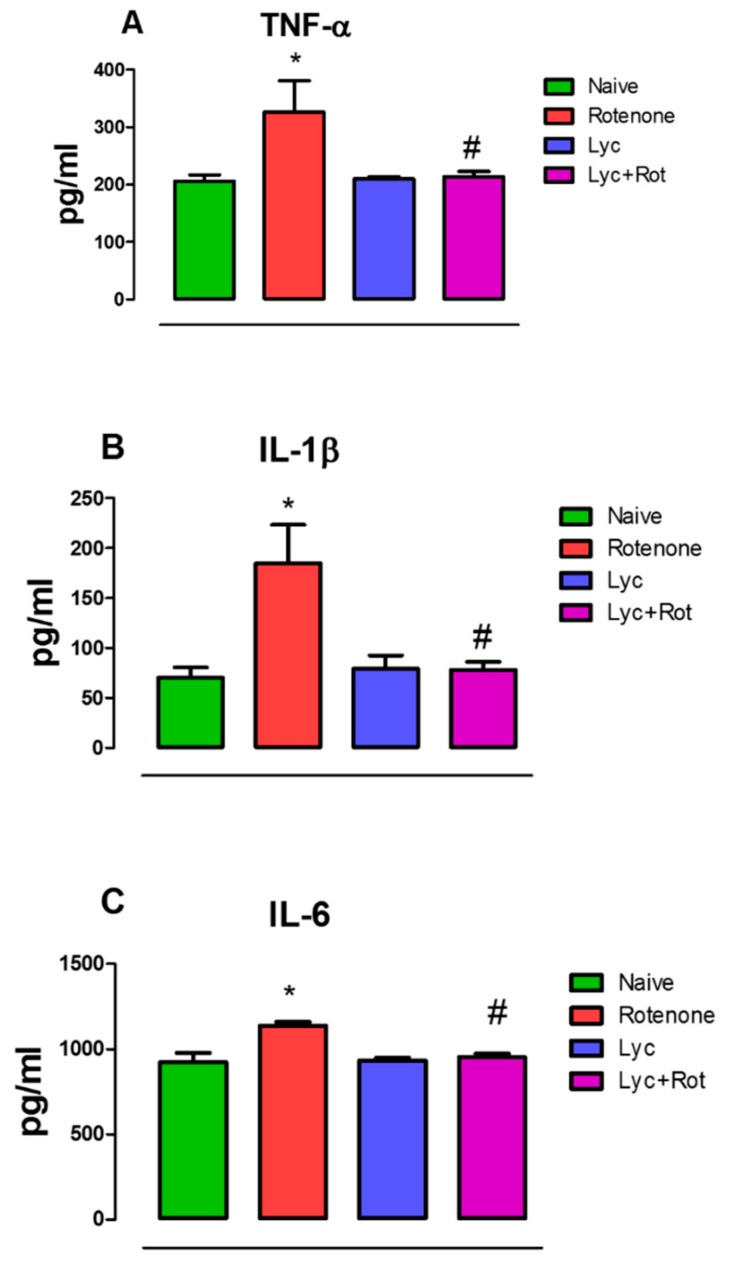
ELISA analysis of TNF-α, IL-1β and IL-6 in experimental animals. Enzyme linked Immunosorbent assay showed that rotenone administration increased the expression of TNF-α (**A**), IL-1β (**B**) and IL-6 (**C**). However, Lyc treatment significantly decreased the expression of pro-inflammatory factors in rotenone-intoxicated animals. Values are expressed as mean ± SEM. * *p* < 0.05 compared to control, ^#^ *p* < 0.05 compared to rotenone-treated group.

Tumor necrosis factor-α, IL-1β and IL-6 are important mediators that activate and propagate neuroinflammation. We therefore examined the levels of these proinflammatory cytokines in the experimental animals. Rotenone administration induced a rise in TNF-α, IL-1β and IL-6 (Figure 3A–C) in the midbrains, compared to our control. Interestingly, Lyc administration to rotenone-treated animals significantly decreased (*p* < 0.05) the levels of pro-inflammatory cytokines when compared to the rotenone group. However, Lyc alone did not show any significant change in the levels of TNF-α, IL-1β and IL-6.

### 2.4. Lyc Offers Neuroprotection by Inhibiting Matrix Metalloproteinase Expression

**Figure 4:**
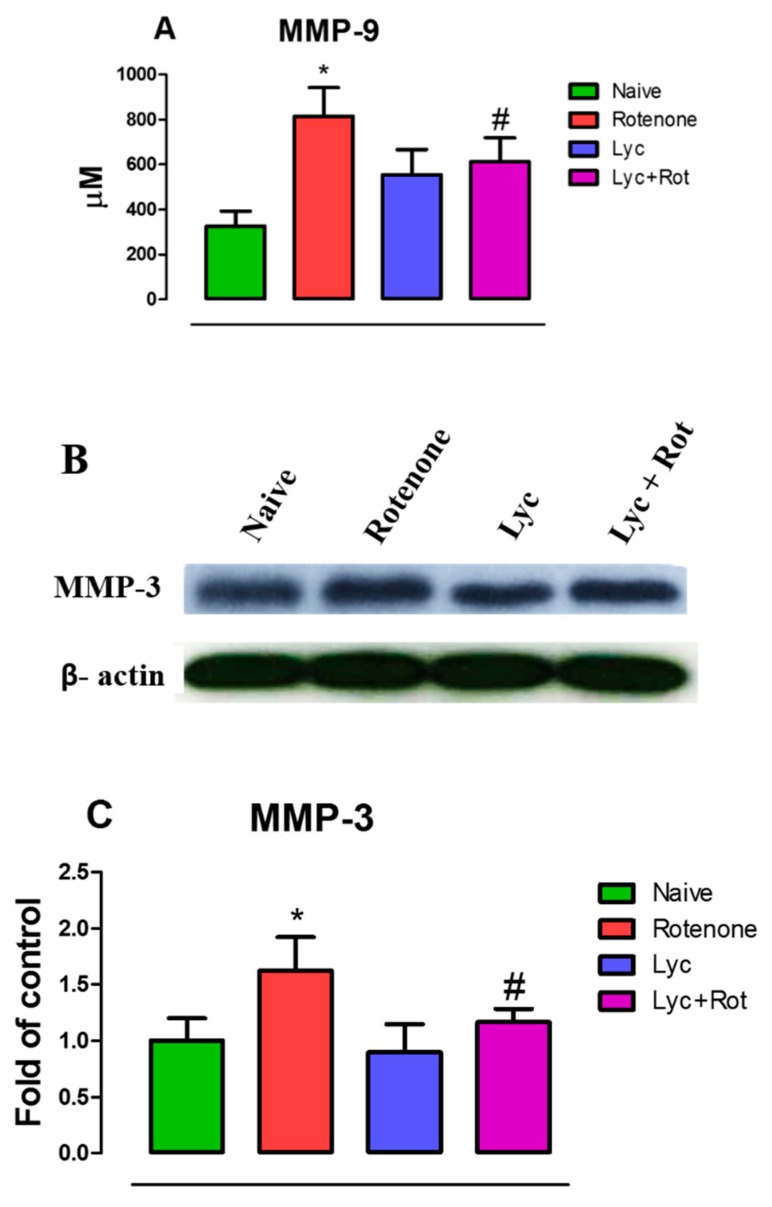
Treatment with Lyc diminished the expression of MMP-9 and MMP-3. Rotenone-treated animals showed a significant increase in matrix metalloproteinases, MMP-9 (**A**) and MMP-3 (**B**). However, treatment with Lyc diminished the expression of MMP-9 and MMP-3 by virtue of its anti-inflammatory action. Histogram (**C**) represents densitometric analysis of western blots, and values are expressed as mean ± SEM. * *p* < 0.05 compared to control, ^#^ *p* < 0.05 compared to the rotenone-treated group.

MMP-3 and MMP-9 play a major role as an initiator of neuroinflammation and in particular, MMP-3 is released from apoptotic dopaminergic neurons. Hence, we assessed if Lyc could diminish MMP expression that may abolish pro-inflammatory response upon rotenone challenge. The ELISA (Figure 4A) and western blotting (Figure 4B) studies showed that rotenone administration significantly (*p* < 0.05) elevated the expression of MMP-9 and MMP-3 compared to controls. Interestingly, Lyc administration suppressed MMP-mediated inflammation by dampening the activities of MMP-9 and MMP-3.

### 2.5. Lyc Exerts Neuroprotection by Modulating Glial Response

**Figure 5:**
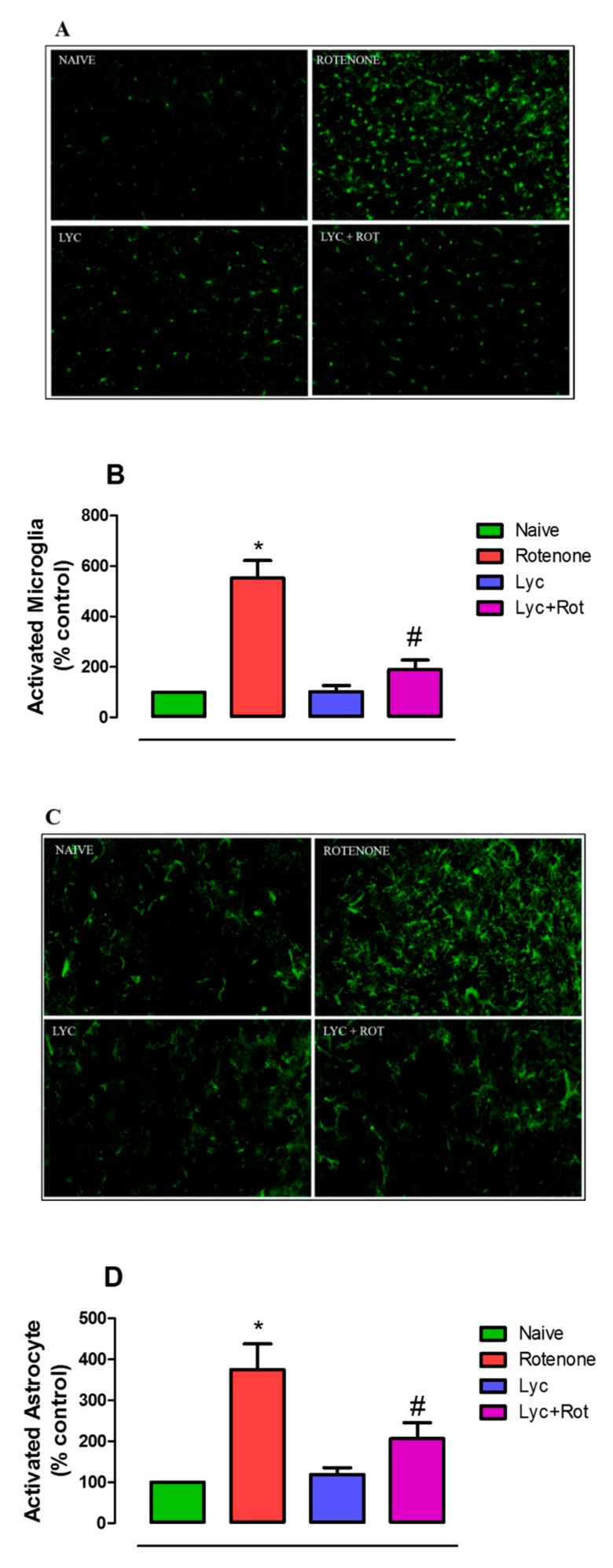
Effect of Lyc on rotenone-induced microglia and astrocyte activation. Immunofluorescent staining of brain tissues with Iba-1 (**A**) and GFAP (**C**) of experimental animals. Picture shows 20 µm thick sections of striatum between different groups. Densitometric assessment of fluorescent intensity was performed to quantify microglia (**B**) and astrocyte (**D**) activation using Image J. Rotenone administration caused profound increase in Iba-1 positive microglia and GFAP positive astrocyte response. Alternatively, Lyc treatment decreased the activation of microglia and astrocyte, as evinced by a decrease in Iba-1 and GFAP positive cells. Values are expressed as mean ± SEM. * *p* < 0.05 compared to control, ^#^ *p* < 0.05 compared to the rotenone-treated group.

Further, to support the above findings, both the microglial and astroglial responses were assessed using immunofluorescent staining. As shown in Figure 5A,C, rotenone administration significantly (*p* < 0.05) enhanced the expression of Iba-1 positive microglia (5 fold) and GFAP-positive astrocytes (3.7 fold), respectively, compared to the control. Whereas, Lyc treatment clearly ameliorated microglial and astrocyte activation in the striatum. Quantification of Iba-1 and GFAP immunoreactivity also showed that Lyc regimen significantly reduced rotenone-induced glial activation (Figure 5B,D).

### 2.6. Effect of Lyc on Alpha-Synuclein Expression

**Figure 6:**
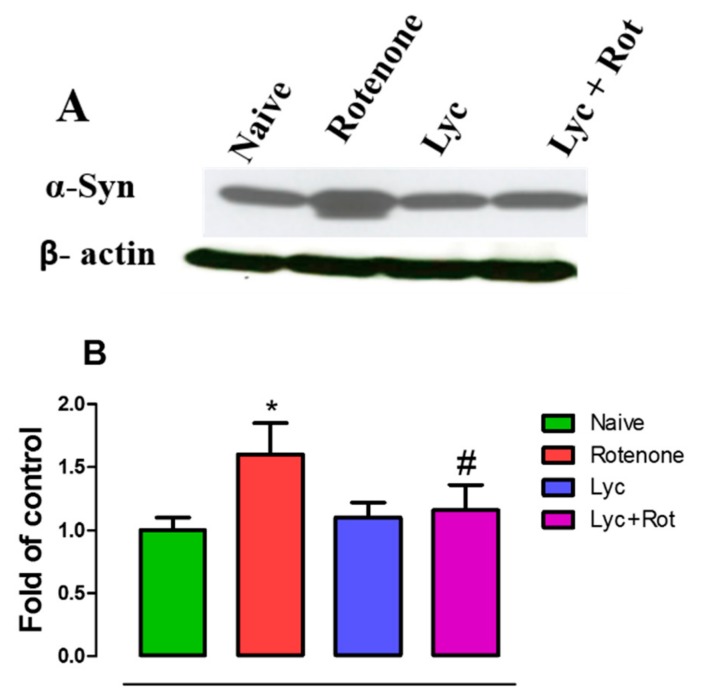
Effect of Lyc in alpha-synuclein expression in control and experimental animals. Lycopodium prevented rotenone-induced alpha-synuclein expression (**A**) in experimental animals. Protein expression were quantified and represented (**B**) as fold of control. Values are expressed as mean ±SEM. * *p* < 0.05 compared to control, ^#^ *p* < 0.05 compared to the rotenone-treated group.

Immunoblotting was performed to evaluate the expression of α-synuclein, as a measure of protein-dependent toxicity in experimental groups. Rotenone injections significantly increased the expression of α-synuclein when compared to control rats (Figure 6). Whereas, treatment with Lyc decreased α-synuclein expression compared to the rotenone group.

### 2.7. Lyc Administration Diminished COX-2 and iNOS Expression

**Figure 7:**
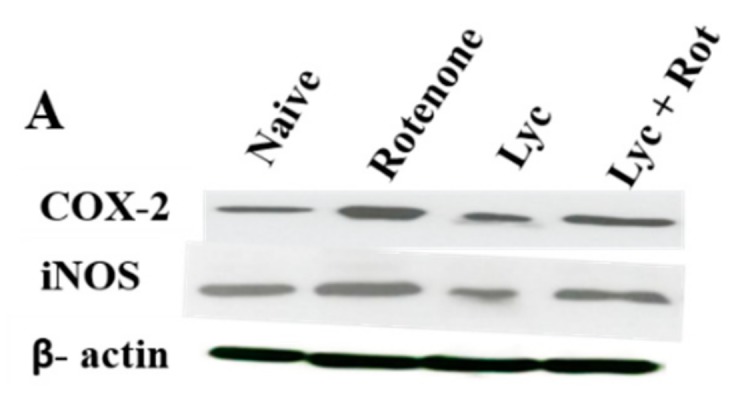

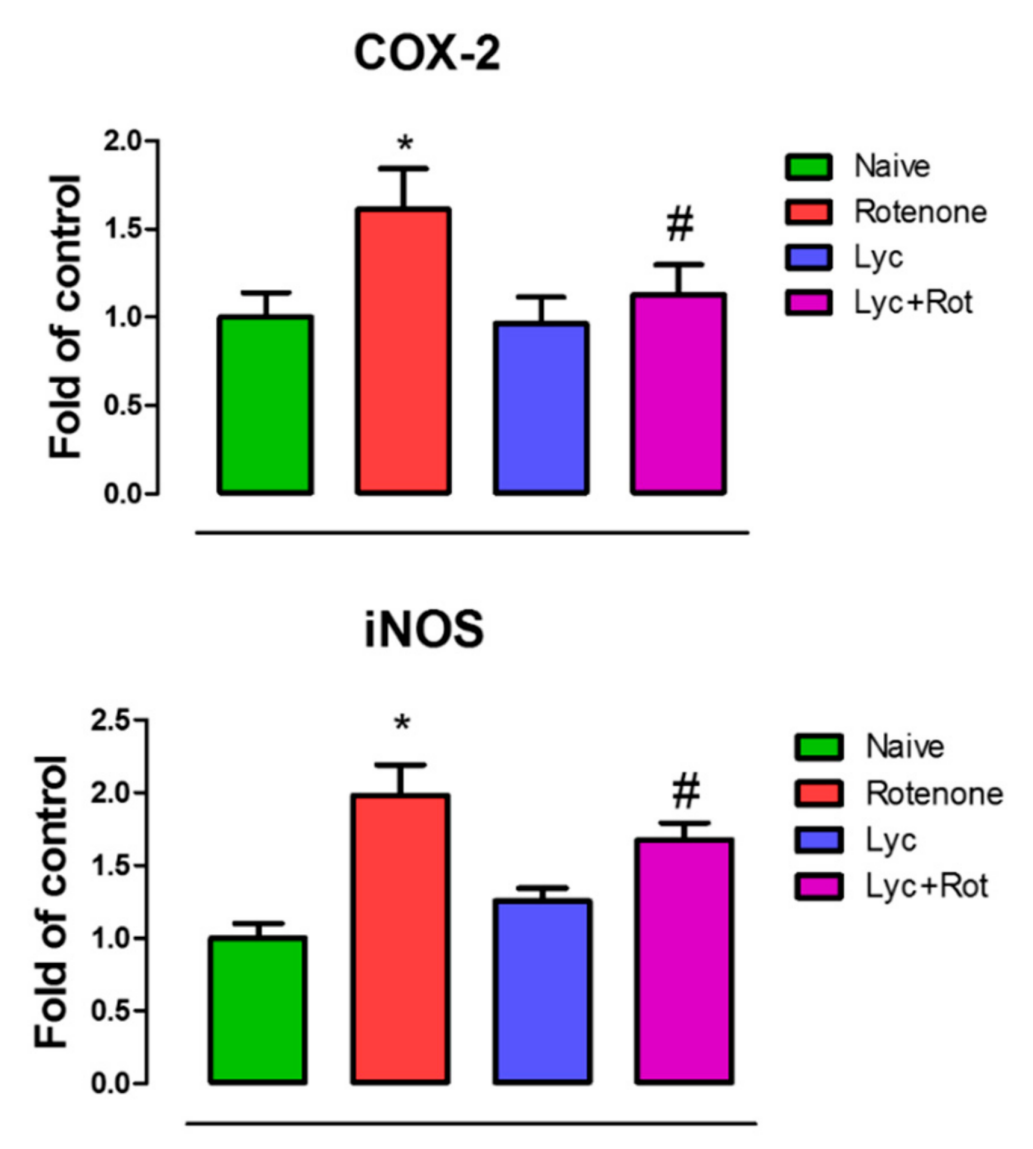
Immunoblotting analysis of Cox-2 and iNOS. Immunoblots of midbrain protein samples probed with Cox-2 and iNOS (**A**). Quantitative analysis of COX-2 and iNOS western blots were shown in histogram B and C, respectively. Values are expressed as mean ± SEM. * *p* < 0.05 compared to control, ^#^ *p* < 0.05 compared to the rotenone-treated group.

Overexpression of Cox-2 and iNOS play an important role in inducing neuroinflammation in the pathogenesis of PD. Hence, we evaluated the effect of Lyc on Cox-2 and iNOS expression using western blotting. As shown in Figure 7, rotenone significantly increased (*p* < 0.05) the expression of Cox-2 and iNOS compared to our control. However, these expression levels were significantly decreased upon treatment with Lyc when compared to rotenone-injected rats.

### 2.8. Lyc Protects Dopaminergic Neurons from Rotenone Induced Neurodegeneration

**Figure 8:**
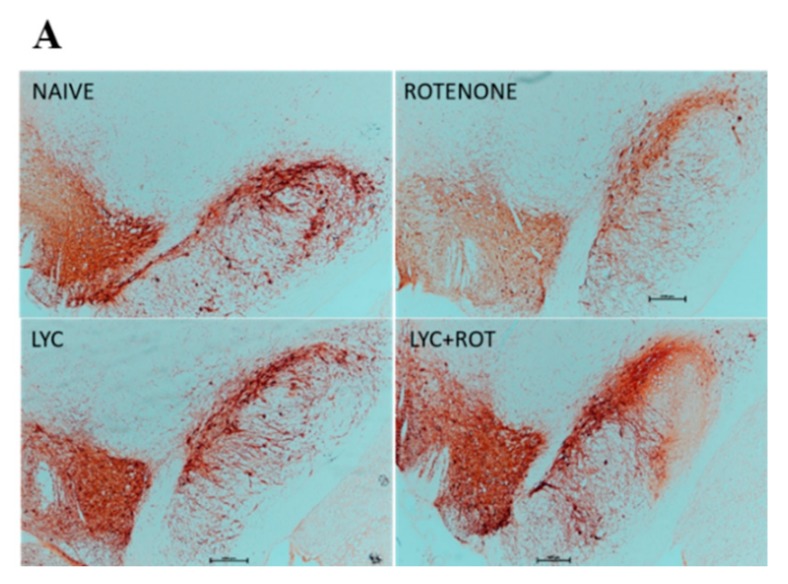

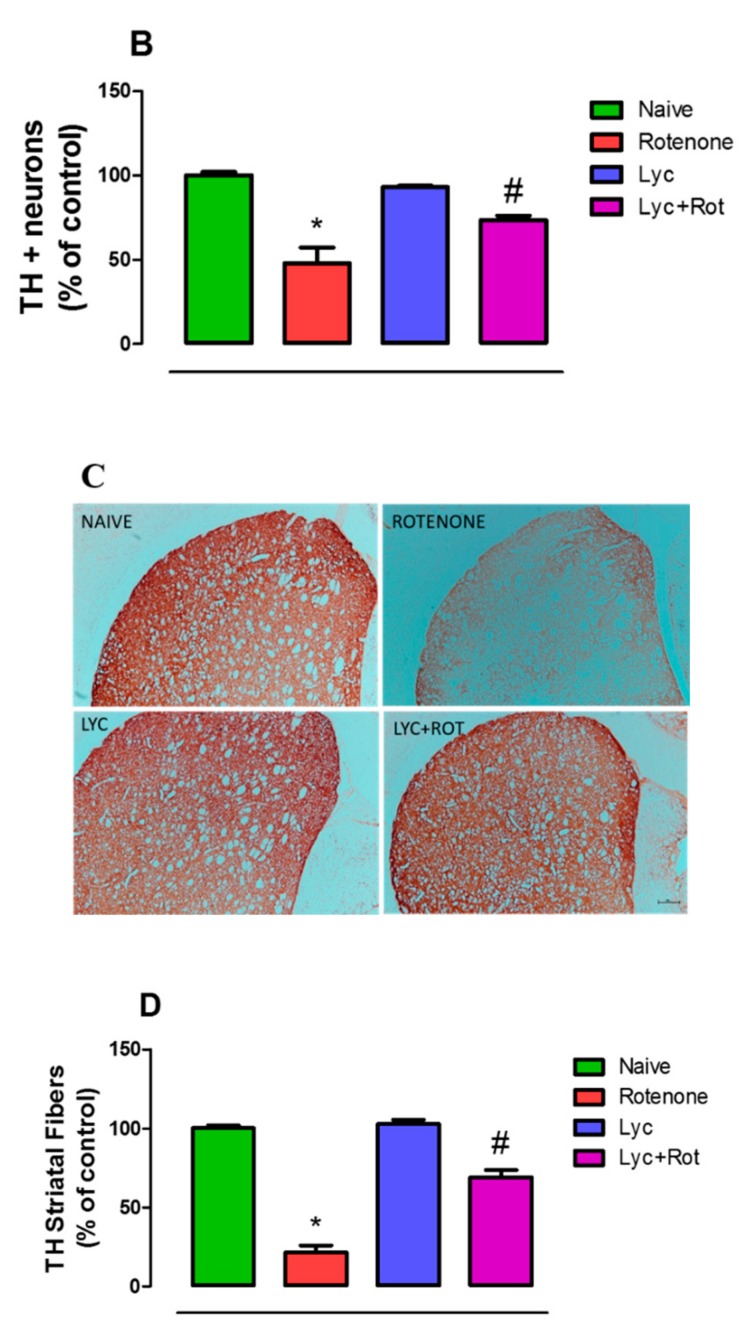
Lyc prevented dopaminergic neurodegeneration. Immunohistochemistry of 20 µm coronal sections with anti-tyrosine hydrolase antibody of substantia nigra (**A**) and striatum (**C**) of experimental animals. Rotenone administration caused the significant loss of both TH+ve neurons and neuronal fibers in the substantia nigra and striatum, respectively. Whereas, administration of Lyc prevented this loss significantly. Quantification of tyrosine hydrolase positive neurons (**B**) and densitometric analysis of tyrosine hydrolase positive neuronal fibers (**D**) were performed using Image J Values are expressed as mean ± SEM. * *p* < 0.05 compared to control, ^#^ *p* < 0.05, compared to the rotenone-treated group.

To further explore whether these anti-inflammatory and anti-oxidant effects of Lyc can protect dopaminergic neurons from rotenone toxicity, we performed Tyrosine Hydroxylase (TH) immunohistochemistry in the brain sections of experimental animals. Analyzing the expression of TH in substantia nigra and striatum are the hallmark evidence in assessing the protective activity of a drug. Thus, TH-ir neurons in SNpc and TH-ir fibers in the striatum were assessed by counting the number of immunoreactive neurons, and by measuring the optical density, respectively. Rotenone-induced neurotoxicity is evidenced by a significant loss of nigral TH+ neurons in SNpc, and a decrease in the intensity of TH-ir fibers in the striatum (Figure 8). However, Lyc treatment to rotenone-treated rats resulted in a significant (*p* < 0.05) increase in the number of nigral dopaminergic neurons that concurs with enhanced intensity of TH-ir fibers in the striatum. There were no significant neuronal losses in control- and Lyc-treated groups.

## 3. Discussion

Though there are various therapies for the treatment of PD, an ideal drug would be the one that not only impedes the progression of dopaminergic neuronal loss, but also rescues the patient from the disease. However, various treatment strategies have been proven beneficial initially, but prolonged treatments resulted in severe side effects [29]. PD is characterized by loss of dopaminergic neurons, however, evidence suggests an early alteration of cholinergic neurotransmission in PD brain.

Hence, anticholinergic medications such as trihexyphenidyl, benztropine, orphenadrine, procyclidine, and biperidenare are used for the symptomatic treatment of PD [30]. A recent report shows that Cholinesterase inhibitors are effective for cognitive impairment in patients with PD, and that it also reduces falling in PD [31]. However, drugs that are used for cholinesterase inhibitory activity are limited, and possess side effects related to cholinergic stimulation in peripheral tissues and the brain. Many literature reports state that oxidative stress-mediated neuroinflammation could be the major culprit, self-propelling neurodegeneration in Parkinson’s disease [32,33,34]. Hence, the search for novel multi-modal drugs that have neuroprotective, neurorestorative and anti-cholinergic capabilities, along with less side effects, would be promising for the treatment of Parkinson’s disease.

Various studies demonstrated that Lycopodium alkaloids, *Lycopodium clavatum* and *Lycopodium thyoides*, possess potent acetylcholinesterase inhibitory and antioxidant effects [35,36]. However, the neuroprotective effect of Lycopodium in an in vivo model has not been studied. In this context, our study was designed to evaluate the neuroprotective potential of Lyc using a rotenone-induced PD rat model. The present study substantiates that Lyc prevents dopaminergic neurodegeneration by impeding lipid peroxidation and enhancing antioxidant response, decreasing pro-inflammatory cytokines (TNF-α, IL-1β, IL-6), and the activities of inflammatory mediators, MMP-9 and MMP-3. The reduction in α-synuclein and a restoration of tyrosine hydroxylase expression in substantia nigra and striatum further supported the neuroprotective effects of Lyc.

Rotenone-induced PD models are largely used to decipher the pathological mechanisms, and to find promising therapeutic drugs [37]. Rotenone is a highly lipophilic insecticide, which accumulates in the mitochondria, and inhibits mitochondrial complex I, ensuing in reactive oxygen species (ROS) production, glutathione depletion and subsequent oxidative stress. Biochemical and post-mortem studies provided strong evidence for the association of hyperoxidation and protein carbonyl formation in PD patients [38]. As noted, any literature reports state that oxidative stress-mediated neuroinflammation could be the major culprit, self-propelling neurodegeneration in Parkinson’s disease [32,33,34]. Hence, agents with strong antioxidant property could be important in PD treatment and prevention.

Zecca et al., (2008) reported that neuromelanin, an oxidation product of dopamine induces neuroinflammation and neurodegeneration in Parkinson’s disease [39]. Various studies have reported the decrease in GSH level, and activities of catalase and SOD in the brains of Parkinson’s patients [40,41]. Our experiments demonstrated that Lyc-enhanced antioxidant defense, by decreasing the levels of Malondialdehyde (MDA) and nitric oxide, and correspondingly increased the levels of vital antioxidants (GSH, SOD and Catalase) in rotenone-treated rats. Our results, proving the antioxidant potential of Lyc, are in line with a previous study of a Lyc-enhanced antioxidant response in aging mice [42]. Lyc has also been reported to protect against cognitive [43], inflammatory [24,25] and neuronal [36,42] damage.

Several reports showed evidence about the role of Matrix metalloproteinases (MMPs) in inflammation-mediated neurodegeneration. MMPs are zinc- and calcium-dependent endopepti-dases, and they play an important role in neurogenesis, axonal guidance, myelogenesis as well as supporting synaptic plasticity, learning and memory [44,45]. Apart from the normal physiological roles, the pathological roles of MMPs are also influenced by oxidative stress [46]. Choi et al., (2003) reported that treatment of primary dopaminergic cells with tetrahydrobiopterin (obligatory cofactor of tyrosine hydroxylase and potent oxidative source) induces MMP-3 toxicity, and this effect is prevented by antioxidants [47]. Various studies have reported enhanced MMP-3 expression in both PD model and PD brain [48,49]. In addition, Choi et al. (2008) reported that intracellular enzymatic activity of MMP-3 is primarily responsible for dopaminergic neurodegeration [50]. Evidence suggests that MMP-3 is activated under stress conditions in dopaminergic neurons, which triggers microglial activation in the extracellular space, which in turn contributes to neuronal damage. However, the inhibition of MMP-3 actually attenuated neuronal death [51]. These studies suggest that MMPs play a major role in initiating neuroinflammation and any resultant dopaminergic neuronal death. Moreover, another important matrix metalloproteinase, MMP-9, is also reported to be elevated in MPTP-induced PD mice [52].

In addition, MMP-3 itself is a self-sufficient player in the activation of microglia and its resultant pro-inflammatory cascade [10]. Hence, we evaluated whether Lyc confers neuroprotection by modulating MMP activities. For this reason, we investigated the expression of MMP-9 and MMP-3 by ELISA and western blotting, respectively. Our results demonstrate that rotenone increased the expression of MMP-9 and MMP-3 that was diminished by Lyc treatment. Subsequently, MMP-3 has been reported to activate microglia, which subsequently release pro-inflammatory cytokines, favor-ing microgliosis [10]. Stimulation of microglia with MMP-3 induces ERK signaling pathways, but not JNK (c-Jun N-terminal protein kinase), which in turn aggravates neuronal apoptosis [11]. Lorenzl et al. (2003) reported that a pharmacological inhibition of MMP-9 protected dopaminergic neuronal loss, and diminished dopamine depletion [52]. Similarly, the same group have reported that MMP-9 is primarily localized in neurons, and the latter report was supported by Annese et al. (2015), where MMP-9 contributes to glial activation and neurodegeneration in both the monkey and mouse MPTP-PD models. Studies using MMP-9-/- mice showed that MPTP administration resulted in less microglial activation and more a dopaminergic survival rate, when compared to wild type animals [53]. All these findings pinpoint that MMPs are key initiator molecules for the onset of neuroinflammation. In line with these findings, our study also explicated that the administration of rotenone enhanced the expression of MMP-9 and MMP-3.

We further evaluated the activities of microglia, astrocyte and proinflammatory cytokine production in rotenone-treated animals. Immunofluorescent studies depicted that rotenone administration-activated microglia and astrocyte resulted in a subsequent release of pro-inflammatory cytokines. However, Lyc administration diminished the expression of Iba-1 positive microglia, GFAP-positive astrocyte and the resultant pro-inflammatory cytokine production.

Post-mortem and experimental evidence have showed that enhanced expression of Inducible nitric oxide synthase (iNOS) and Cox-2 potentiates inflammatory cascade [3,54]. Cox-2 influences neuroinflammation via the production of pro-inflammatory prostaglandins as well as ROS. iNOS is typically expressed in physiological conditions, but excess pro-inflammatory cytokines enhance the production of iNOS, resulting in the production of nitric oxide and subsequent peroxynitrite. Expression of iNOS is a characteristic marker of microglia and astrocyte activation [55]. In addition, iNOS overexpression inhibits neuronal respiration that results in depolarization, glutamate release and excitotoxicity [56]. Earlier reports indicated that rotenone intoxication caused a significant increase in the expression of Cox-2 and iNOS in the rat brain [57]. Similarly, our experiments showed that rotenone administration markedly increased Cox-2 and iNOS expression. Whereas, administration of Lyc diminished these expressions, which supports the anti-inflammatory property of Lycopodium. 

Our studies and reports from other groups have demonstrated that oxidative stress promotes alpha-synuclein aggregation and subsequent neurodegeneration [58,59,60]. Moreover, a study by Xu et al. (2002) reported that overexpression of wild type alpha-synuclein resulted in ROS accumulation and apoptosis in human fetal dopaminergic neurons. Conversely, overexpressing α-synuclein in non-dopaminergic human cortical neurons results in protection. Supplementary to this hypothesis, wild type α-synuclein co-exists with anti-apoptotic 14-3-3 protein in dopaminergic cells, which could explain the enhanced neuronal susceptibility to apoptosis. Conclusively, in dopaminergic neurons, α-synuclein triggers ROS, apoptosis that could be effectively inhibited by vitamin E, an antioxidant [61]. Choi et al. (2011) reported that α-synuclein and MMP-3 were co-localized in the Lewy bodies of post-mortem brain PD subjects [49]. Moreover, enhanced expression of α-synuclein and exposure of amyloid beta to SK-N-BE cells induced MMP-3 expression [48]. Various reports demonstrate that α-synuclein is cleaved by MMP-3, generating different C-terminal truncated peptides that cause stronger dopaminergic neuronal death [48,49]. These reports suggest that there is a strong correlation between MMP-3, α-synuclein and neurodegeneration. Hence, we measured the expression of wild type alpha-synuclein upon rotenone administration. Supporting previous reports [62,63], our results demonstrated that rotenone injections caused a significant increase in α-synuclein expression. However, the administration of Lyc diminished the expression when compared to rotenone-treated groups. 

The mechanism behind this might be that Lyc could upregulate the expression of various heat shock proteins (HSP-70, HSP-40 and HSP-27) which are reported to reduce alpha-synuclein aggregation [64,65].

Finally, we wanted to evaluate whether the neuroprotective mechanisms of Lyc could lead to the protection of dopaminergic neurons, which was analyzed by tyrosine hydroxylase immunohistochemistry in substantia nigra and striatum. Tyrosine hydroxylase (TH) is a rate-limiting enzyme that is involved in the conversion of tyrosine to 3,4-dihydroxyphenylalanine (DOPA), which is further converted to dopamine by the aromatic amino acid decarboxylase. Various studies have demonstrated that TH activity is progressively diminished following the death of dopaminergic neurons in substantia nigra in PD patients [66,67]. Concordant with these reports, our results also showed that rotenone administration significantly caused dopaminergic neuronal death as evinced by less TH-ir neurons and diminished TH-ir fibers in the striatum. One of the mechanisms underlying this toxicity might be that rotenone promotes neuronal death by microglial phagocytosis [68]. In support of this study, previous reports state that apoptosis of dopaminergic neurons releases MMP-3 which in turn triggers microglial activation, and elevated nitric oxide activates MMP-9, which results in the disruption of the extracellular matrix, and leads to cell detachment and apoptosis of neurons [50,69]. In addition, since the nigral brain region has high microglial density, obviously, dopaminergic neurons could be instantly affected because of microglial activation [70]. Based on various reports, Kim et al. (2011) concluded that MMP-3 could be a potential therapeutic target since it contributes to neurodegeneration via multiple pathways [70].

Grounded on our experimental results, we report that Lyc conferred neuroprotection against rotenone-induced PD by inhibiting ROS formation, α-synuclein expression, MMP orchestrated glial activation, and subsequent neurodegeneration. Thus, we speculate that Lyc, owing to its pleiotropic mechanisms, could be further developed as a possible therapeutic drug for the treatment of Parkinson’s disease.

## 4. Materials and Methods

### 4.1. Drugs and Chemicals

Monoclonal mouse anti-α-synuclein was purchased from BD biosciences, San Jose, CA. Polyclonal rabbit anti-cyclooxygenase-2 (Cox-2), anti-inducible nitric oxide synthase (iNOS) and anti-glial fibrillary acidic protein (GFAP) were procured from Sigma-Aldrich, St. Louis, MO, USA. Anti-ionized calcium-binding adaptor molecule-1 (Iba-1) was purchased from Wako chemicals, Richmond, USA. Polyclonal rabbit anti-tyrosine hydrolase antibody was obtained from Merck, Germany. Antibodies against MMP-2 was purchased from Abcam, Cambridge, MA, USA. Alexa Flour 488 conjugated goat anti-rabbit secondary antibodies were purchased from Thermo Fischer Scientific, Waltham, MA, USA. Biotinylated secondary goat anti-rabbit antibody was purchased from Jackson Immunoresearch, West grove, USA. Lycopodium extract, Rotenone and other analytical grade reagents were procured from Sigma Aldrich, St. Louis, MO, USA. The biochemical assays were performed using commercially-available kits.

### 4.2. Experimental Animals and Procedure

Healthy male Wistar rats (11–12 months old) weighing 280–300 g were obtained from the Animal Research Facility, College of Medicine and Health Sciences, United Arab Emirates University (UAEU). The animals were kept in polypropylene cages in air-conditioned room (23 ± 2 °C, relative humidity of 45–55%) with a 12 h light/dark cycle and with an ad libitum access to food and water. The experimental procedures were strictly followed according to the guidelines approved by the Institutional Animal Ethics Committee of the United Arab Emirates University (ERA-2018-5785) to reduce animal suffering, and use a minimum number of animals in the study, without compromising the objectives to be tested.

Study Design: Rotenone (ROT) stock solution (50X) was initially prepared by dissolving ROT in in dimethyl sulfoxide (DMSO) and further diluted in myglol to get a final concentration of 2.5 mg/mL. Intraperitoneal injections of ROT (2.5 mg/kg) once daily for 4 weeks have been well demonstrated to mimic the neurodegeneration similar to PD in humans. The regimen used in this study for the induction of Parkinsonism has been well standardized in our laboratory in concurrence with previous report (33). Pharmacological effects of Lyc in vivo were examined using four treatment groups (n = 15/group).

Lyc was administered orally (50 mg/kg) once daily for four weeks. After 30 min, ROT was administered intraperitoneally at a dose of 2.5 mg/kg. Experimental animals were randomly distributed into four experimental groups, each containing eight rats. Group I served as control and received an intraperitoneal injection of the vehicle. Group II received a rotenone injection daily for four weeks. Group III received oral administration of Lyc 30 min before their rotenone injection. Group IV received Lyc alone for four weeks.

### 4.3. Tissue Preparation for Biochemical Studies

After 4 weeks of treatment, pentobarbital (40 mg/kg body weight) was used to anesthetize the animals followed by cardiac perfusion (0.01 M phosphate-buffered saline at pH 7.4) to eliminate the blood. Brains were immediately removed, and both hemispheres were separated on ice. One hemisphere was postfixed for 48 h in 4% paraformaldehyde solution and then PFA was exchanged with 10% sucrose solution for three successive days (4 °C) prior to immunohistochemical studies. Midbrain and striatum were isolated from the other half hemisphere and snap frozen in liquid nitrogen for biochemical and inflammatory studies.

### 4.4. Biochemical Studies

Biochemical analysis was carried out using midbrain samples from each group which were homogenized in KCl buffer (Tris–HCl 10 mM, NaCl 140 mM, KCl 300 mM, ethylenediaminetetraacetic acid 1 mM, Triton-X 100 0.5%, pH 8.0) supplemented with phosphatase and protease inhibitor. Post-mitochondrial supernatant was collected by centrifuging midbrain homogenates at 14,000× *g* for 20 min (4 °C) for biochemical studies.

### 4.5. Estimation of Lipid Peroxidation

Lipid peroxidation was determined by assessing Malondialdehyde (MDA) levels, following manufacturer’s instruction provided in the MDA detection kit procured from North West Life Science (Vancouver, WA, USA). Briefly, the calibrators or samples (250 μL) were incubated in the presence of thiobarbituric acid (250 μL) and acid reagent followed by rigorous vortexing. The mixture was incubated for 1 h at 60 °C and then centrifuged at 10,000× *g* for 2–3 min. The spectra was measured at 532 nm using spectrophotometer and results were expressed as μm MDA/mg protein.

### 4.6. Estimation of Reduced Glutathione

The levels of glutathione in brain homogenates were measured using Sigma’s glutathione assay kit (Sigma-Aldrich Chemie GmbH, Steinheim) as per the manufacturer’s instruction. Brain homogenates were deproteinized with 5% 5-sulfosalicylic acid solution, followed by centrifugation to eliminate precipitated protein, and supernatant was used to estimate GSH. Standards or samples (10 μL) were incubated with 150 μL of working mixture (assay buffer + 5,5′-dithiobis (2-nitrobenzoic acid) + GSH reductase) for 5 min in 96-well plates. Fifty microliters of Diluted NADPH solution was added into each well and mixed thoroughly. The absorbance of the samples was measured at 412 nm using the microplate reader with the kinetics for 5 min and results were expressed as μm GSH/mg protein.

### 4.7. Estimation of the Activities of Antioxidant Enzymes

The influence of treatment on antioxidant enzymes [superoxide dismutase (SOD) and catalase (CAT)] in each group was estimated using Cayman assay kits (Cayman Chemicals Company, Ann Arbor, MI, USA) following manufacturer’s instructions. Catalase activity was measured by adding 20 μL of standards or samples and 30 μL of methanol to the assay buffer (100 μL) in 96-well plates. Twenty microliter hydrogen peroxide was added and the mixture was incubated for 20 min at room temperature to initiate the reaction. After incubation, potassium hydroxide (30 μL) was used to stop the reaction, followed by subsequent addition of catalase purpald (30 μL) and catalase potassium periodate (10 μL). The reaction plate was incubated at room temperature for 5 min on shaker, and absorbance was read at 540 nm. The CAT activity was expressed as nmol/min/mg protein. For SOD measurement, 10 μL of standards or samples were added followed by xanthine oxidase (20 μL) into each well of 96-well plates to initiate the reaction. The reaction mixture was mixed for few seconds and incubated (covered) for 30 min at room temperature. Absorbance was measured at 450 nm using microplate reader. The activity of SOD was expressed as units/mg protein.

### 4.8. Estimation of Nitrite Levels

Nitrite levels in the experimental samples were analyzed using a commercially available R&D kit (Minneapolis, MN, USA). Briefly, the nitrite standard or samples (50 µL) were added onto a 96-well plate (supplied by the manufacturer) along with the reaction diluent (50 µL) Then, 50 µL of Griess reagent was added and mixed by gentle tapping. The plate was incubated at RT for 10 min and the absorbance was read at 540 nm (microplate reader). Nitrite levels were reported as µmol/mg protein.

### 4.9. Estimation of Proinflammatory Cytokines and MMP-3 by ELISA Assays

Enzyme linked immunosorbent (ELISA) assays were performed to evaluate the levels of tumor necrosis factor-alpha (TNF-α), interleukin-1β (IL-1β), interleukin-6 (IL-6) and MMP-3 using commercially available kits (Bio Source International Inc., CA, USA). Briefly, 100 μL of capture antibody (diluted) was added onto the plates and kept overnight at room temperature. The wells were then aspirated and washed using wash buffer (0.05% Tween 20 in PBS 0.01 M pH 7.4). Reagent diluent [1% bovine serum albumin in PBS (300 μL)] was added onto the plate and blocked for 1 h. After blocking, the plates were washed with wash buffer and standards or samples (100 μL) were added. The plate was incubated at RT for 2 h. Detection antibody (100 μL) was added and the plate was again incubated for 2 h at RT. The well was then exchanged with 100 μL of working solution (1:200, streptavidin horseradish peroxidase) and the plate was further incubated. After 20 min, the wells were exchanged with substrate solution (100 μL) and incubated further for 20 min. Finally, the stop solution [2N H2SO4, (50 μL)] was added into each well. Experimental plate was read immediately at 450 nm using microplate reader and results were reported as pg/mg protein.

### 4.10. Immunofluorescence Staining of GFAP and Iba-1

The activation of microglia and astroglia in the striatum was performed by immunofluorescence staining analyzing Iba-1 and GFAP, respectively. Briefly, sections were washed with PBS twice and incubated with blocking solution (10% normal goat serum in PBS 0.3% Triton-X 100) for 1 h at RT. After 1 h, blocking reagent was removed and the sections were incubated with primary antibodies against GFAP (1:1,000) and Iba-1 (1: 1000) overnight at 4 °C. After incubation, the sections were washed thrice and incubated with corresponding fluorescent secondary antibody (Alexa 488 anti-rabbit) for 1 h at room temperature. Later, the stained sections were washed and mounted using Vectastain fluorescent mounting media. The images were taken under fluorescent microscope EVOS FL (Thermo Fisher Scientific) and representative image is presented in the results.

### 4.11. Assessment of Activated Astrocytes and Microglia in the Striatum

Minimum of striatal three sections (similar level) of each animal were used to investigate astrocytes and microglia activation. Activated astrocytes and microglia from each section were analysed from randomly chosing different fields (three) of equal area by using the Image J software (NIH, Bethesda, MD, USA) following the method of McCloy et al. 2014 [71]. Briefly, an outline was drawn around the region of interest and area, circularity, mean fluorescence was measured, along with different adjacent background readings. The total corrected cellular fluorescence (TCCF) was calculated using the formula, TCCF = integrated density − (area of selected cell × mean fluorescence of background readings). All readings were measured by an observer blind to the treatment conditions to guarantee the lack of bias. Results were represented as percentage of control.

### 4.12. Western Blot Analysis of MMP-3, α-synuclein, COX-2 and iNOS

Western blotting was performed to evaluate the expression of MMP-3, α-synuclein, COX-2 and iNOS in striatum of experimental animals. Briefly, the tissues were homogenized in RIPA buffer (with protease and phosphatase inhibitor). The homogenates were centrifuged at 12,000 rpm for 20 min at 4 0C. Supernatents containing equal amounts of protein (35 μg) were separated in 10% sodium dodecyl sulphate-polyacrylamide gel electrophoresis. Separated proteins were then transferred onto polyvinylidene difluoride membrane (PVDF) and incubated overnight with primary antibodies against COX-2 (1:1000), iNOS (1:500), MMP-3 (1:600), α-synuclein (1:750), at 4 °C. The membranes were washed and incubated with horseradish peroxidase-conjugated corresponding secondary antibodies and the immunoreactive bands were visualized using Enhanced Chemiluminescence Pico Kit (Thermo Fisher Scientific). The blots were stripped and re-probed for β-actin (1:5,000; monoclonal mouse; EMD Millipore, Billerica, MA, USA) as a loading control. Intensity of the bands was quantified (n = 3) using Image J software (NIH, Bethesda, MD, USA).

### 4.13. Immunohistochemistry for Tyrosine Hydroxylase (TH) Expression

Tyrosine hydroxylase (TH) immunohistochemistry was performed to investigate the neuroprotective property of Lyc against dopaminergic neurodegeneration induced by rotenone. Briefly, coronal brain sections (14 μm) were cut striatum and substantia nigra (SNc) levels using a cryostat (Leica, Wetzlar, Germany) and the sections were washed twice with 0.01 M of PBS, pH 7.4. Then, the sections were incubated with blocking reagent (10% normal goat serum in PBS containing 0.3% Triton-X 100) for 1 h at RT. Following blocking, the sections were incubated with anti-TH primary polyclonal rabbit antibody (1:500) overnight at 4 °C. Later, sections were washed thrice and incubated for 1 hr at RT with biotinylated secondary anti-rabbit (1:1000) antibody. After washing, the sections were incubated with avidin–biotin complex (Vector Laboratories Ltd. Burlingame, CA, USA) followed by 3,3′ diaminobenzidine (DAB). Finally, the sections were coverslipped using DPX mounting medium and the slides were viewed under a light microscope (Olympus, Hamburg, Germany).

### 4.14. Assessment of TH-ir Dopaminergic Neurons and TH-ir Dopamine Nerve Fibers Loss

To evaluate TH immunoreactive (TH-ir) neurons in the SNc area, three different levels of the medial terminal nucleus region were counted, and the average value was represented as percentage of control. Striatal fiber loss was quantified by measuring the optical density of TH-ir dopaminergic fibers in the striatum using Image J software. Optical density of TH-ir fibers from each section (three sections/rat) within the striatum was analyzed for each animal and an average of three areas were calculated and reported as a percentage. Optical density of overlying cortex was used as a background measure and subtracted from the value generated from the striatum. Investigator blind to the experimental groups carried out counting of TH-ir neurons and optical density of the TH-ir fibers.

### 4.15. Protein Estimation

Protein concentration from each sample was measured using Pierce BCA protein assay kit (Thermo Fisher Scientific) following the manufacturer’s instruction.

### 4.16. Statistical Analyses

Data were expressed as the mean value±SEM. To calculate the statistical significance between various groups one-way analysis of variance followed by Tukey’s test was performed using SPSS 12 software. In all the experiments, the data was considered statistically significant at *p* < 0.05.
